# Reevaluating the Salty Divide: Phylogenetic Specificity of Transitions between Marine and Freshwater Systems

**DOI:** 10.1128/mSystems.00232-18

**Published:** 2018-11-13

**Authors:** Sara F. Paver, Daniel Muratore, Ryan J. Newton, Maureen L. Coleman

**Affiliations:** a Department of the Geophysical Sciences, University of Chicago, Chicago, Illinois, USA; b School of Freshwater Sciences, University of Wisconsin Milwaukee, Milwaukee, Wisconsin, USA; Argonne National Laboratory

**Keywords:** 16S rRNA, SAR11, aquatic ecology, aquatic microbiology, biogeography, environmental transitions, microbial ecology, tag sequencing

## Abstract

Marine and freshwater microbial communities are phylogenetically distinct, and transitions between habitat types are thought to be infrequent. We compared the phylogenetic diversity of marine and freshwater microorganisms and identified specific lineages exhibiting notably high or low similarity between marine and freshwater ecosystems using a meta-analysis of 16S rRNA gene tag-sequencing data sets. As expected, marine and freshwater microbial communities differed in the relative abundance of major phyla and contained habitat-specific lineages. At the same time, and contrary to expectations, many shared taxa were observed in both habitats. Based on several metrics, we found that *Gammaproteobacteria*, *Alphaproteobacteria*, *Bacteroidetes*, and *Betaproteobacteria* contained the highest number of closely related marine and freshwater sequences, suggesting comparatively recent habitat transitions in these groups. Using the abundant alphaproteobacterial group SAR11 as an example, we found evidence that new lineages, beyond the recognized LD12 clade, are detected in freshwater at low but reproducible abundances; this evidence extends beyond the 16S rRNA locus to core genes throughout the genome. Our results suggest that shared taxa are numerous, but tend to occur sporadically and at low relative abundance in one habitat type, leading to an underestimation of transition frequency between marine and freshwater habitats. Rare taxa with abundances near or below detection, including lineages that appear to have crossed the salty divide relatively recently, may possess adaptations enabling them to exploit opportunities for niche expansion when environments are disturbed or conditions change.

**IMPORTANCE** The distribution of microbial diversity across environments yields insight into processes that create and maintain this diversity as well as potential to infer how communities will respond to future environmental changes. We integrated data sets from dozens of freshwater lake and marine samples to compare diversity across open water habitats differing in salinity. Our novel combination of sequence-based approaches revealed lineages that likely experienced a recent transition across habitat types. These taxa are promising targets for studying physiological constraints on salinity tolerance. Our findings contribute to understanding the ecological and evolutionary controls on microbial distributions, and open up new questions regarding the plasticity and adaptability of particular lineages.

## Abstract (precis)

The distribution of microbial diversity across environments yields insight into processes that create and maintain this diversity as well as potential to infer how communities will respond to future environmental changes. We integrated data sets from dozens of freshwater lake and marine samples to compare diversity across open water habitats differing in salinity. Our novel combination of sequence-based approaches revealed lineages that likely experienced a recent transition across habitat types. These taxa are promising targets for studying physiological constraints on salinity tolerance. Our findings contribute to understanding the ecological and evolutionary controls on microbial distributions, and open up new questions regarding the plasticity and adaptability of particular lineages.

## INTRODUCTION

Phylogenetic relationships of organisms within and across ecosystems can provide insight into the evolutionary history of lineages and how evolution might proceed into the future. Microorganisms in the water columns of freshwater and marine ecosystems provide a unique juxtaposition. On one hand, these habitats share common features of pelagic lifestyles like free-living and particle-associated niches (1), potential for interactions with phytoplankton (2), and opportunities for diverse photoheterotrophic organisms, including aerobic anoxygenic phototrophs (3) and rhodopsin-containing bacteria (4, 5). However, salinity preference is considered a complex trait involving many genes and complex cellular integration (6, 7), suggesting that transitions between high and low salinity are difficult from a genetic perspective. Consistent with this idea, microbial communities from saline environments are compositionally distinct from those inhabiting nonsaline environments (8, 9). Salinity-induced shifts in microbial beta diversity have been observed in studies of marine-to-freshwater gradients in many systems, including the Baltic Sea (7, 10), Columbia River Estuary system (11), and Antarctic lakes that have become progressively less saline since becoming isolated from the sea (12). These observations of ecosystem-specific diversity support the current paradigm that transitions between marine and freshwater ecosystems are infrequent, despite many ecological similarities (13).

Environmental sequence data provide support for a “salty divide” separating marine and freshwater microbial assemblages. From a phylogenetic perspective, each clade that contains both marine and freshwater representatives includes at least one transition where a common ancestor gave rise to a daughter lineage able to survive and proliferate in a new salinity environment (13). Transitions that occurred recently are expected to result in highly similar molecular sequences recovered from marine and freshwater systems while transitions that occurred in the distant past are expected to yield habitat-specific diversification—clades that are only observed in one habitat type or the other—and a greater sequence divergence between marine and freshwater representatives (13). Prior work using phylogenetic patterns concluded that transitions between marine and freshwater environments are infrequent and most transition events occurred a long time ago in evolutionary terms (13, 14). For example, Logares and colleagues (14) found that within the abundant alphaproteobacterial SAR11 group, freshwater representatives belonged exclusively to a single subclade, called LD12, implying a single salinity transition from a marine ancestor to this freshwater lineage. Besides LD12, there are a number of microbial lineages that appear to be unique to freshwater lakes (15, 16), suggesting that these lineages do not readily colonize other habitat types. Notably, for freshwater lineages that are found in multiple habitats, the secondary habitat is most often terrestrial, not marine (16), consistent with the idea that marine-freshwater transitions are especially difficult.

Difficulty in detecting transitions between marine and freshwater systems may contribute to the paradigm that transitions occur infrequently. Detecting a transition requires sufficiently abundant extant descendants. Most immigrant cells are expected to go extinct locally due to ecological drift, just as most mutations are lost from a population due to genetic drift (17). The probability of an immigrant avoiding extinction due to ecological drift, like a mutation avoiding genetic drift, depends on the degree of selective advantage. For example, in populations of Escherichiacoli (∼3 × 10^7^ cells [18]), a mutation conferring a 10% advantage appears an average of five times before it is established compared to a mutation with a 0.1% advantage which would need to appear 500 times to avoid extinction by drift (19). In addition to overcoming ecological drift, the degree of selective advantage for cells migrating between marine and freshwater habitats would need to be strong enough to overcome any salinity-based disadvantages. Microorganisms that become established must also achieve sufficiently high population abundances to be reliably detected by current sequencing methods. As amplicon sequencing data sets accumulate from an increased diversity of environments and library size increases, our ability to detect transitions improves.

Here we revisit classic questions concerning divisions between marine and freshwater microorganisms by comparing 16S rRNA V4 region amplicon sequences from available marine and freshwater data sets. This meta-analysis is timely given the accumulation of sequence data sets from diverse aquatic environments, including large lakes such as the Laurentian Great Lakes that historically have been underrepresented in sequence databases. These large lakes, sometimes referred to as inland seas, are in some ways more similar to the open oceans than to previously studied small lakes (20). Given their size, they are less influenced by their catchment than smaller lakes and experience oceanic-type physical processes, including strong currents and upwelling (21). Although lakes are generally more productive than the open oceans, parts of the Laurentian Great Lakes are extremely oligotrophic, rivaling the ocean gyres in terms of phosphorus limitation and productivity (22–25). Given these features, we speculated that the Great Lakes would be more likely than small lakes to harbor lineages recently descended from marine ancestors. At a minimum, we reasoned that expanding within-habitat diversity in our comparative analysis would improve the robustness of our conclusions between habitat types. Our specific objectives were to (i) compare the phylogenetic diversity of marine and freshwater microorganisms and (ii) identify lineages that have a comparatively high or low degree of sequence similarity between marine and freshwater ecosystems. These lineages may represent targets for exploring physiological and molecular barriers to salinity tolerance, and for identifying novel strategies for overcoming these barriers.

(This article was submitted to an online preprint archive [26].)

## RESULTS

### Marine and freshwater communities have distinct taxonomic composition.

**FIG 1:**
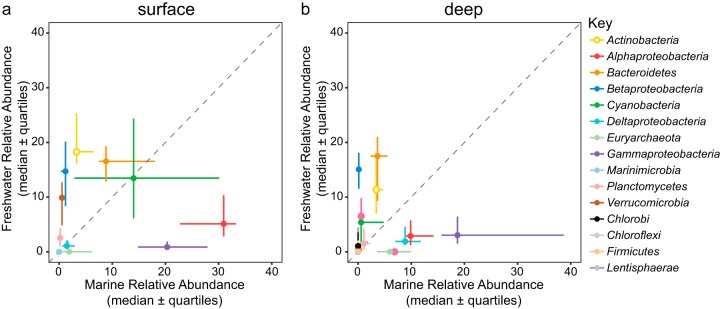
Median relative abundance of phyla/proteobacterial classes in freshwater and marine samples collected from surface (a) and deep (b) waters. The deepest hypolimnion (below thermocline) sample collected from stratified lakes and marine samples collected at depths >75m were classified as “deep” samples. Diagonal lines indicate a 1:1 relationship.

We first asked whether marine and freshwater communities were compositionally distinct in our combined data set, consistent with previous studies. To this end, we compared the abundances of phyla (classes for *Proteobacteria*), orders, and families between marine and freshwater samples. At the phylum level, *Alphaproteobacteria*, *Gammaproteobacteria*, *Euryarchaeota*, and *Marinimicrobia* had significantly higher relative abundances in marine systems while *Betaproteobacteria* and *Verrucomicrobia* had higher relative abundances in freshwater systems (Fig. 1). At finer taxonomic levels, we found that orders, and especially families, showed a positive correlation between relative abundance in freshwater and fold enrichment in freshwater versus marine samples, and vice versa; in other words, the most abundant freshwater families also appeared to be highly specific to freshwater, while numerous other families have similar abundances in marine and freshwater systems (see Fig. S1 in the supplemental material).

10.1128/mSystems.00232-18.1 FIG S1 Log_10_ fold change as a function of log_10_-transformed median relative abundances in each habitat plotted for orders (a, b) and families (c, d) in freshwater (a, c) and marine (b, d) systems. A solid gray line is plotted at a log_10_ fold change of 0, indicating that marine and freshwater median relative abundances are equal. Dotted gray lines are plotted at log_10_ fold changes of 1 and −1, which correspond to median abundance in one habitat being 10× higher than the median abundance in the other habitat. Download FIG S1, EPS file, 0.5 MB. Copyright © 2018 Paver et al. 2018 Paver et al. This content is distributed under the terms of the Creative Commons Attribution 4.0 International license.

We next took a taxon-level approach, using minimum entropy decomposition (MED [27]) to cluster sequences into taxonomic units (i.e., MED nodes) and UniFrac distances to compare assemblages of marine and freshwater nodes. Consistent with previous studies, we found that marine and freshwater assemblages were phylogenetically distinct (Fig. S2; weighted UniFrac PerMANOVA, *F* = 23.6, *R*^2^ = 0.24, *P* < 0.001, df = 75; unweighted UniFrac PerMANOVA, *F* = 41.8, *R*^2^ = 0.36, *P* < 0.001, df = 75). This result is robust even with our expanded data set, suggesting that environmental factors such as nutrient availability and temperature are secondary to salinity in driving overall community composition (Fig. S2b).

10.1128/mSystems.00232-18.2 FIG S2 Principal coordinate analysis of unweighted UniFrac distances between marine and freshwater assemblages characterized by 16S rRNA V4 gene sequences (a). The same ordination color coded by nutrient classification and labeling a group of outlier freshwater samples as those collected from dystrophic bog lakes (b). Download FIG S2, EPS file, 0.5 MB. Copyright © 2018 Paver et al. 2018 Paver et al. This content is distributed under the terms of the Creative Commons Attribution 4.0 International license.

### Phylogenetic distance between marine and freshwater taxa.

We next asked whether particular phyla, orders, and families tended to include closely related marine and freshwater representatives more often than others, reflecting more recent habitat transitions in these groups. Using UniFrac distances between marine and freshwater taxa (MED nodes) as a metric, calculated either pairwise between samples or together with all samples pooled, we found distances generally fell between 0.75 and 0.90 for each phylum (Fig. S3A). For individual orders and families, marine-freshwater distances spanned a greater range (Fig. S3; Table S1). Together, these results indicate that closely related marine and freshwater taxa can be found in most phyla, but they are not distributed uniformly across orders and families within those phyla; rather, some orders and families tend to be enriched in instances of closely related marine and freshwater taxa and therefore in putative recent transitions. For example, gammaproteobacterial families *Chromatiaceae* and *Vibrionaceae* and actinobacterial families PeM15 and *Mycobacteriaceae* had the smallest unweighted UniFrac distances (<0.55) between marine and freshwater taxa, suggesting that marine and freshwater lineages tend to be more closely related in these groups. In contrast, *Hydrogenophilaceae* (*Betaproteobacteria*) and KI89A (*Gammaproteobacteria*) each had UniFrac distances of 1.00 (Table S1), indicating that marine and freshwater lineages we detected within these families were completely distinct phylogenetically.

10.1128/mSystems.00232-18.3 FIG S3 Median unweighted UniFrac distances (bars indicate range) between pairs of freshwater and marine samples compared to unweighted UniFrac distance between combined freshwater and combined marine samples for each phylum and proteobacterial class (a). Unweighted UniFrac distances between combined freshwater and combined marine samples for orders (b) and families (c) within each phylum/class. Phyla, classes, orders, and families are included in the analysis if they occurred in at least 3 freshwater and 3 marine samples and contained at least 5 nodes. Download FIG S3, EPS file, 0.3 MB. Copyright © 2018 Paver et al. 2018 Paver et al. This content is distributed under the terms of the Creative Commons Attribution 4.0 International license.

10.1128/mSystems.00232-18.9 TABLE S1 UniFrac distance calculated between marine and freshwater sequences for each family. Download Table S1, PDF file, 0.04 MB. Copyright © 2018 Paver et al. 2018 Paver et al. This content is distributed under the terms of the Creative Commons Attribution 4.0 International license.

**FIG 2:**
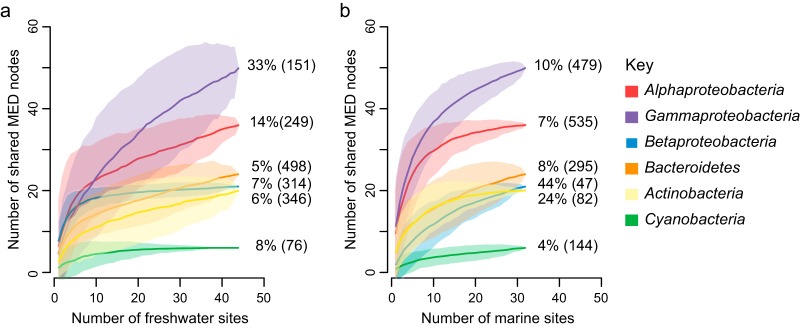
Species accumulation curves for taxonomic groups that contain shared marine and freshwater MED nodes as the number of freshwater sites included in the analysis increases (a) and as the number of marine sites included in the analysis increases (b). The percentage of sequences shared between habitats with all sites analyzed is included to the right of each curve; the total number of MED nodes within each group in freshwater and marine habitats, respectively, is indicated in parentheses.

At the finest taxonomic level, MED nodes, we observed 171 total shared MED nodes, i.e., nodes detected in at least one marine sample and one freshwater sample (Table 1; Fig. S4). For some phyla, our observations of shared taxa have saturated, while we expect to detect new shared taxa in other phyla with greater sampling effort (Fig. 2; Fig. S5). *Betaproteobacteria* and *Gammaproteobacteria* showed the largest increases in the proportion of shared nodes as more marine and freshwater sites were sampled, respectively (Fig. S5). The pronounced increase in the proportion of betaproteobacterial shared nodes as more marine (but not freshwater) sites were analyzed indicates that nodes commonly observed in freshwater are sporadically detected in marine systems, and vice versa for *Gammaproteobacteria*. *Gammaproteobacteria* contained the most shared nodes, which accounted for 10% and 33% of total gammaproteobacterial nodes observed in marine and freshwater systems, respectively. *Alphaproteobacteria* contained the second highest number of shared nodes, accounting for 7% and 14% of total alphaproteobacterial nodes observed in marine and freshwater systems, respectively.

**TABLE 1:** Genera containing at least two shared MED nodes

Phylum/class	Order	Family	Genus^*b*^	No. of shared nodes
*Actinobacteria*	*Acidimicrobiales*	OM1_clade	“*Ca.* Actinomarina”	2
*Actinobacteria*	*Acidimicrobiales*	Sva0996^*a*^	Sva0996^*a*^	2
*Actinobacteria*	*Corynebacteriales*	*Mycobacteriaceae*	*Mycobacterium*	2
*Actinobacteria*	*Micrococcales*	*Microbacteriaceae*	“*Ca.* Aquiluna”	4
*Actinobacteria*	PeM15	PeM15	PeM15	4
*Bacteroidetes*	*Flavobacteriales*	*Cryomorphaceae*	*Fluviicola*	5
*Bacteroidetes*	*Sphingobacteriales*	*Chitinophagaceae*	*Sediminibacterium*	2
*Bacteroidetes*	*Sphingobacteriales*	NS11-12^*a*^	NS11-12^*a*^	3
*Cyanobacteria*	Subsection I	Family I	*Synechococcus*	3
*Marinimicrobia*	SAR406 clade	SAR406 clade	SAR406 clade	4
*Alphaproteobacteria*	*Caulobacterales*	*Caulobacteraceae*	*Brevundimonas*	4
*Alphaproteobacteria*	*Rhizobiales*	*Methylobacteriaceae*	*Methylobacterium*	3
*Alphaproteobacteria*	*Sphingomonadales*	*Sphingomonadaceae*	*Novosphingobium*	2
*Alphaproteobacteria*	*Sphingomonadales*	*Sphingomonadaceae*	*Sphingobium*	3
*Alphaproteobacteria*	*Sphingomonadales*	*Sphingomonadaceae*	*Sphingomonas*	3
*Betaproteobacteria*	*Burkholderiales*	*Burkholderiaceae*	*Ralstonia*	2
*Betaproteobacteria*	*Burkholderiales*	*Comamonadaceae*	*Aquabacterium*	2
*Deltaproteobacteria*	SAR324 clade	SAR324 clade	SAR324 clade	3
*Gammaproteobacteria*	*Alteromonadales*	*Alteromonadaceae*	*Marinobacter*	2
*Gammaproteobacteria*	E01-9C-26^*a*^	E01-9C-26^*a*^	E01-9C-26^*a*^	2
*Gammaproteobacteria*	*Oceanospirillales*	*Oceanospirillaceae*	*Pseudohongiella*	4
*Gammaproteobacteria*	*Oceanospirillales*	OM182 clade	OM182 clade	2
*Gammaproteobacteria*	*Oceanospirillales*	SAR86 clade	SAR86 clade	2
*Gammaproteobacteria*	*Pseudomonadales*	*Moraxellaceae*	*Acinetobacter*	5
*Gammaproteobacteria*	*Pseudomonadales*	*Pseudomonadaceae*	*Pseudomonas*	3
*Gammaproteobacteria*	*Vibrionales*	*Vibrionaceae*	*Vibrio*	2
*Euryarchaeota*	*Thermoplasmatales*	Marine group II	Marine group II	2
*Thaumarchaeota*	Unknown order	Unknown family	“*Ca.* Nitrosopumilus”	2

a Marine group.

b *Ca*., *Candidatus*.

10.1128/mSystems.00232-18.4 FIG S4 Phylogenetic trees of select phyla and proteobacterial classes: (A) *Alphaproteobacteria*, (B) *Actinobacteria*, (C) *Betaproteobacteria*, (D) *Chloroflexi*. External ring colors indicate the habitats where MED nodes were detected in the data set: freshwater (green), marine (blue), both (orange). Notable clades are indicated by colored wedges. Download FIG S4, EPS file, 1.6 MB. Copyright © 2018 Paver et al. 2018 Paver et al. This content is distributed under the terms of the Creative Commons Attribution 4.0 International license.

10.1128/mSystems.00232-18.5 FIG S5 Fraction of freshwater nodes shared with marine systems as a function of freshwater sites sampled (a) and fraction of marine nodes shared with freshwater systems as a function of marine sites sampled (b). Download FIG S5, EPS file, 0.4 MB. Copyright © 2018 Paver et al. 2018 Paver et al. This content is distributed under the terms of the Creative Commons Attribution 4.0 International license.

### Direct sequence-level comparisons reveal variation across phyla.

To quantify differences among phyla in their marine-freshwater transition history, we sought to compare all sequences in our data set using a fundamental metric—sequence identity—without assigning sequences to MED nodes or operational units. For each phylum, we constructed an all-versus-all distance matrix using pooled sequences from all samples, and clustered this matrix using every possible sequence identity threshold (to form 99.6% clusters, 99.3%, 99.0%, etc., given an amplicon size of 290 bp). Then, for all pairwise combinations of one marine and one freshwater sample (i.e., all marine-freshwater sample pairs), we identified the highest cluster threshold at which the two samples shared sequences in the same cluster (Fig. S6). We interpreted this threshold as a phylum-specific proxy for time since the most recent marine-freshwater transition: for example, finding 100% identical sequences in a marine and freshwater sample pair would imply a very recent transition event, whereas a cluster threshold of only 70% identity would imply a deep branching split into exclusively marine and freshwater clades (in other words, sequence clusters at all cutoffs greater than 70% would consist of exclusively marine or freshwater members). We summarized this identity threshold for each major phylum (class for *Proteobacteria*) and across all pairwise sample comparisons.

10.1128/mSystems.00232-18.6 FIG S6 As sequence identity cutoff value increases, more taxa are shared between marine-freshwater sample pairs. Decrease in Jaccard index between marine-freshwater sample pairs (unshared taxa)/(shared taxa) with increasing sequence similarity cutoff values for *Alphaproteobacteria* and *Verrucomicrobia* (a). Example phylogenetic trees are shown for groups with high sequence similarity (b) and low sequence similarity (c) between marine (blue) and freshwater (green) samples. Download FIG S6, EPS file, 0.9 MB. Copyright © 2018 Paver et al. 2018 Paver et al. This content is distributed under the terms of the Creative Commons Attribution 4.0 International license.

**FIG 3:**
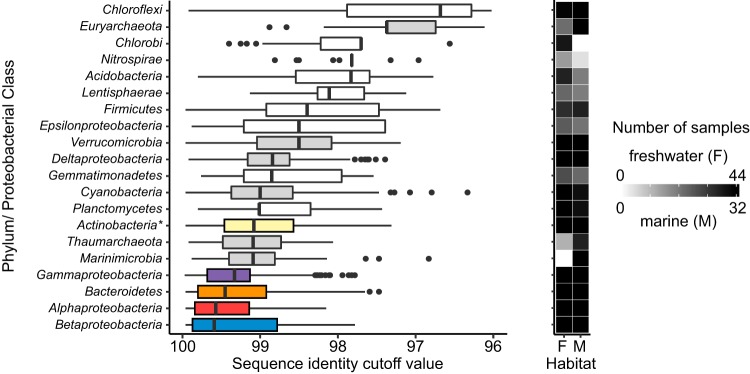
Maximum sequence identity threshold (i.e., finest-scale resolution) at which pairs of marine and freshwater samples share common taxa. Box plots indicate the median, quartiles, and range of values observed for all marine-freshwater sample pairs. Colored boxes indicate phyla/proteobacterial classes that contain 5 or more shared MED nodes while gray boxes indicate groups that contain 1 to 3 shared MED nodes. The heatmap to the right illustrates the number of freshwater (F) and marine (M) samples containing representatives of each phylum/proteobacterial class. *, *Actinobacteria* cutoff values were calculated with a preclustered data set (see Fig. S5 for comparison of all groups using a preclustered data set).

Using this approach, we found that most phyla contained shared taxa at identity thresholds >99% for at least some pairs of marine and freshwater samples (Fig. 3). This result is not due to a few particular samples that tended to share taxa more frequently. Instead, it suggests that recent marine-freshwater transitions are phylogenetically and geographically widespread. At the same time, however, we also found substantial variation across phyla: some phyla showed widespread evidence for recent transitions across all sample pairs, while other phyla showed sporadic or no evidence for recent transitions (Fig. 3; Fig. S6 and S7). Within the *Alpha*- and *Betaproteobacteria*, for example, sequences typically were shared between marine and freshwater samples with 96% identity (median value for all pairwise sample comparisons). At the other extreme, no *Nitrospirae* sequences were found to be shared between marine and freshwater samples at >89% identity. In addition, sequences from *Chloroflexi*, *Euryarchaeota*, and *Chlorobi* were rarely shared between marine and freshwater samples at >77% identity.

10.1128/mSystems.00232-18.7 FIG S7 Minimum sequence identity threshold (i.e., finest-scale resolution) at which pairs of marine and freshwater samples share common taxa. In contrast to Fig. 2, this analysis used a data set where sequences were preclustered to allow up to 2 base pair differences within a sequence group. Box plots indicate the median, quartiles, and range of values observed for all marine-freshwater sample pairs. A heat map illustrates the number of freshwater (F) and marine (M) samples containing representatives of each phylum/proteobacterial class. Download FIG S7, EPS file, 0.4 MB. Copyright © 2018 Paver et al. 2018 Paver et al. This content is distributed under the terms of the Creative Commons Attribution 4.0 International license.

### Genome-wide evidence for recent marine-freshwater transitions in the SAR11 group.

**FIG 4:**
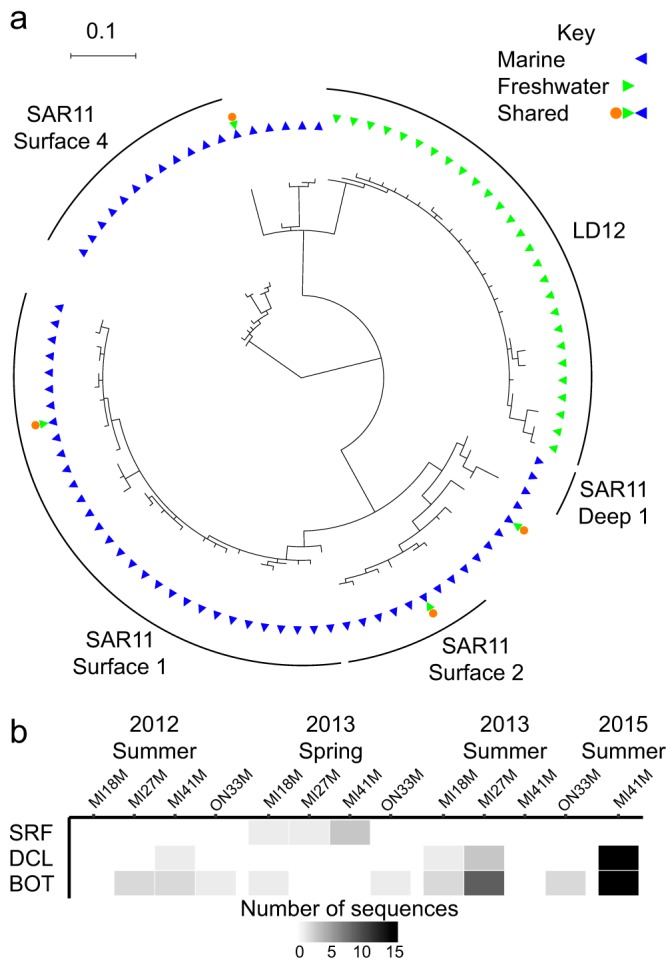
Observations of non-LD12 SAR11. (a) 16S rRNA V4 region gene tree constructed using representative sequences from each SAR11 node. The first ring indicates whether nodes were found only in marine (blue) or freshwater samples (green) while the second ring indicates nodes that are shared across habitat types (orange). (b) Number of non-LD12 SAR11 clade sequences detected at four stations (MI18M, MI27M, MI41M, ON33M) and three depths (SRF, surface; DCL, deep chlorophyll maximum layer; BOT, near-bottom) on Lakes Michigan and Ontario.

Given these overall phylogenetic patterns of marine-freshwater transitions, we sought to illustrate the implications for a single taxonomic group as a case study. We chose to focus on the SAR11 group of *Alphaproteobacteria* because representatives of this group are extremely abundant in both marine and freshwater systems, providing ample data in both habitat types. Further, this group has been the focus of a prior study which found that all freshwater SAR11 fell within the LD12 clade, reflecting a single major transition (14). Unexpectedly, our analysis detected several instances where non-LD12 SAR11 taxa (MED nodes) were found in freshwater: clades surface 1 and surface 2 were observed in a humic lake, an estuarine clade was observed in a Tibetan Plateau lake, and an unclassified SAR11 clade was observed in the Laurentian Great Lakes (Fig. 4a). Like many of the shared nodes in our data set, the marine (i.e., non-LD12) SAR11 shared nodes were detected at very low abundances in freshwater. One of the shared nodes was observed in the Laurentian Great Lakes, a system where we have been collecting microbial community data for several years, so we expanded our search for non-LD12 SAR11 to our larger data set, beyond the eleven samples initially included in the meta-analysis. Based on data sets acquired with the 515F/806R primer set, which is known to bias against the SAR11 clade (28), this non-LD12 SAR11 node accounted for 1 to 15 sequences out of an average of approximately 75,000 sequences per sample (Fig. 4b). The distribution of this node appears to be restricted to the hypolimnion during summer stratification; we detected it in surface samples only during spring sampling when the lakes were mixing.

**FIG 5:**
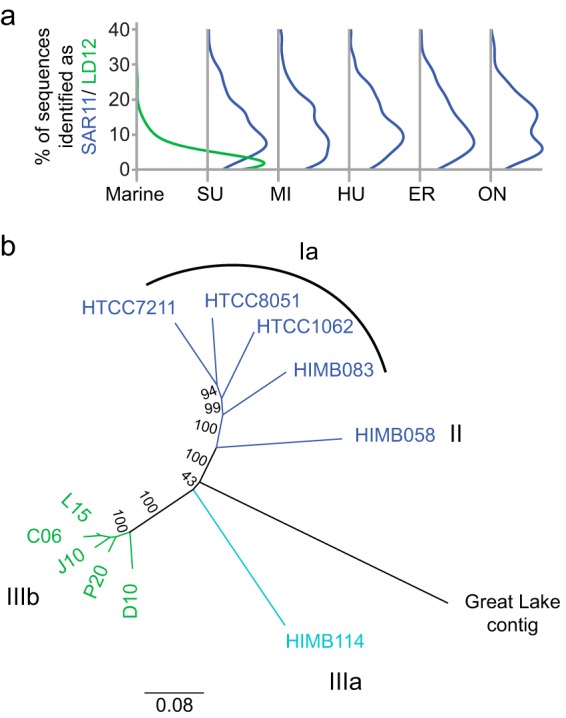
Metagenomic evidence for non-LD12 SAR11 in the Laurentian Great Lakes. (a) Percentage of classified reads identified as LD12 (green) in an open ocean sample (Marine), compared to marine (i.e., non-LD12) SAR11 (blue) in each of the five Laurentian Great Lakes (SU, Superior; MI, Michigan; HU, Huron; ER, Erie; ON, Ontario). Ridge plots present the distribution of identified reads across all protein clusters with greater than 100 reads classified as SAR11 or LD12 at a likelihood value of 0.95. (b) Neighbor-joining consensus tree of 1.2-kb nucleotide sequences from the protein cluster identified as COG2609 (pyruvate dehydrogenase complex, dehydrogenase E1 component). Strain names are colored based on phylogenetic classification within the SAR11 clade: green, LD12 sequences from group IIIb; light blue, group IIIa, sister group to IIIb; medium blue, all other marine SAR11 clades included in the analysis (Ia, II); black, a contig assembled from the Lake Erie metagenome. Consensus support values (%) are indicated on branches.

We reasoned that if these typically marine SAR11 lineages were truly inhabiting the Great Lakes, we would expect to find genome-wide evidence beyond 16S rRNA. To test this, we extracted metagenome reads from the Great Lakes representing *Pelagibacterales* core genes and classified these reads into SAR11 clades using pplacer. We then quantified the relative abundance of classical freshwater LD12 and marine (non-LD12) clades based on this metagenome approach. For each metagenome analyzed, 59% of sequences within a typical (median) protein cluster could be classified as either LD12 or marine (non-LD12) SAR11 at a likelihood of 0.95. Of the classified sequences, 11 to 12% (median value across all protein clusters) were classified as marine SAR11 in each of the Great Lakes samples; for comparison, 98% were classified as marine SAR11 in a marine sample from the Tara Oceans expedition (Fig. 5; Fig. S8). Across protein clusters, the fraction of sequences classified as marine (non-LD12) SAR11 was much more variable for Great Lakes samples (range 0 to 57%, interquartile range 6 to 18%) than for the marine sample (range 76 to 100%, interquartile range 96 to 99%). We identified 199 protein clusters with more than 5% of sequence reads classified as marine (non-LD12) SAR11 in all five Great Lakes metagenomes, suggesting that a substantial fraction of Great Lakes SAR11 cells resemble their marine cousins throughout their genomes, not just at the level of the 16S rRNA gene.

10.1128/mSystems.00232-18.8 FIG S8 Neighbor-joining consensus tree of nucleotide sequences (a) and nucleotide and protein alignment (b) of partial genes from the protein cluster identified as COG2609 (pyruvate dehydrogenase complex, dehydrogenase E1 component). Strain names are colored based on phylogenetic classification within the SAR11 clade: green, LD12 sequences from group IIIb; light blue, group IIIa, sister group to IIIb; blue, all other marine SAR11 clades included in the analysis (Ia, II); black, a sequence read from the Lake Erie metagenome identified by pplacer as marine SAR11. Consensus support values (%) are indicated on tree branches (a). Vertical boxes have been added to the alignment to indicate amino acids where the Great Lakes sequence contains shared characters with a subset of included strains. Download FIG S8, JPG file, 0.8 MB. Copyright © 2018 Paver et al. 2018 Paver et al. This content is distributed under the terms of the Creative Commons Attribution 4.0 International license.

## DISCUSSION

Our meta-analysis sought to use newly available data sets, as well as new analyses, to revisit the paradigm of infrequent transitions between marine and freshwater habitats and identify lineages that may cross the salinity divide with higher or lower frequency than average. We found that marine and freshwater microbial communities were phylogenetically distinct at various phylogenetic resolutions, consistent with the conclusion of Lozupone and Knight (8) and Thompson and colleagues (9) that salinity is the major environmental determinant separating free-living bacteria from different environments. Further, our finding of higher relative abundances of *Betaproteobacteria* and *Actinobacteria* in lakes and higher relative abundances of *Alphaproteobacteria* and *Gammaproteobacteria* in marine systems corresponds with taxonomic comparisons made using metagenomic sequence data sets (29) as well as previous observations using 16S rRNA sequences (16, 30).

### Taxonomic groups with comparatively high transition frequency.

We used multiple approaches to compare relative marine-freshwater transition frequency across phylogenetic groups, based on two key assumptions: (i) the more similar two sequences are, the more recently a common ancestor transitioned between marine and freshwater habitat types, and (ii) each clade containing shared taxa, including every shared node, encompasses at least one transition between habitat types. From these analyses, a coherent picture has begun to emerge. Most phyla contain at least a few instances of recent transitions, but these recent transitions are not evenly distributed. *Alphaproteobacteria*, *Betaproteobacteria*, *Bacteroidetes*, *Gammaproteobacteria* and *Actinobacteria* were inferred to have the most frequent transitions between marine and freshwater systems based on the number of shared MED nodes, i.e., taxa detected in both marine and freshwater systems. Using our direct sequence comparison method, these phyla also exhibited high (>90%) average identity between nearest marine and freshwater representatives (Fig. 3). These phyla encompass a variety of aquatic lifestyles, from small streamlined SAR11 cells that harvest low-molecular-weight dissolved organic matter (31) to particle-attached *Bacteroidetes* with the ability to degrade polymers and genes for gliding motility (32). More frequent transitions in these phyla may stem, in part, from their abundance: *Alphaproteobacteria* and *Gammaproteobacteria* are two of the most abundant phyla in marine systems while *Actinobacteria*, *Betaproteobacteria* and *Bacteroidetes* dominate freshwater lakes, increasing the probability of dispersal across habitat types.

In addition, particular lineages within these phyla may have evolved traits that facilitate successful colonization across a range of environments and salinities (e.g., through lateral gene transfer [33]). These phyla all include organisms capable of photoheterotrophy, which may enable microbial cells to persist until conditions arise that allow population expansion (3, 34–38). A number of aquatic bacterial strains have also been identified as salinity generalists, including representatives of the *Comamonadaceae* (*Betaproteobacteria*), *Pseudomonadaceae* (*Gammaproteobacteria*), *Vibrionaceae* (*Gammaproteobacteria*), and *Pseudoalteromonadaceae* (*Gammaproteobacteria*) (39); all four of these families contained shared MED nodes in our meta-analysis (13, 4, 5, and 1 node[s], respectively). Notably, *Actinobacteria* had lower sequence similarity between pairs of marine and freshwater samples and contained fewer shared nodes than the other four major groups. Below-average growth rates (40) and the dependence of some actinobacterial lineages on other bacteria (41) may contribute to apparent differences in the ability of *Actinobacteria* and other abundant taxa to transition between marine and freshwater systems.

### Insights from the SAR11 group.

Initial evidence pointed to a single marine-freshwater transition in the evolutionary diversification of SAR11, based on the observation that all SAR11 detected in freshwater systems belonged to the LD12 clade while no marine sequences were identified as LD12 (14). Recent findings, including our work, have begun to blur this picture. The first indication that non-LD12, marine-like SAR11 inhabit lakes came from a recently reconstructed partial genome classified as SAR11 subtype I/II from Lake Baikal (42). Here, we detected distinct marine (non-LD12) lineages of SAR11 in each of three lake systems: a humic lake in northern Wisconsin, a Tibetan Plateau lake, and the Laurentian Great Lakes. The same non-LD12 SAR11 node was detected in Lakes Michigan and Ontario across multiple years, depths, and stations within each lake, suggesting an established population in this system. Using phylogenetic placement of metagenome reads, we found further evidence for non-LD12 SAR11 in the Great Lakes. Most metagenome reads could not be unambiguously classified to a particular clade, which could indicate that we lack a closely related genome representative. The reads that were classified fell into clades Ia and IIIa, a sister group of LD12 (also known as clade IIIb) commonly found in brackish environments (10, 43, 44); they were not classified with the partial genome from Lake Baikal, implying distinct non-LD12 lineages in these two large-lake ecosystems. Together these findings provide robust evidence that non-LD12 SAR11 inhabit freshwater habitats.

As ecological data accumulate, the challenge becomes reconciling the distributions of specific lineages with their evolutionary history. Comparative genomics suggests that the LD12 lineage descended from a marine ancestor that lost particular genes related to osmolyte uptake, consistent with the ecological distribution of LD12 cells in freshwater and low-salinity brackish environments but not in higher-salinity marine environments (45, 46). Members of the LD12 clade are also distinguished from their marine cousins by carbon metabolism pathways (47, 48), though how these changes are related to freshwater adaptation remains unclear. Our observations of additional SAR11 lineages in inland lakes (i.e., non-LD12), as well as the recovery of a non-LD12 partial genome from Lake Baikal, raise a number of questions. Do these SAR11 lineages also possess specific genome adaptations to freshwater, and are these adaptations the same as or similar to those acquired by LD12? Such adaptations might include gene gains and losses (33, 46, 48), and also a freshwater-like distribution of protein isoelectric points (i.e., fewer acidic proteins, more basic proteins) as observed in the Lake Baikal partial genome (42, 49). Furthermore, are the global dominance of LD12 and the relative obscurity of other SAR11 lineages in freshwater due to specific genome features or chance (e.g., LD12 arrived first and filled available niche space)? Is there potential for future freshwater population expansions for these lineages? Insight into these questions may come from whole-genome sequences from these new freshwater lineages, as well as physiological studies of cultured isolates.

### Detection limits and overlooked diversity.

Organisms with abundances at or near the detection limits of current sequencing practices are frequently removed from analyses that exclude sequences below a specified abundance threshold (27). However, populations with low representation in sequencing libraries may have unintuitively large census population sizes in a system. A population with a density of one cell per ml has a population size of billions of cells in a one-meter-depth layer of a small lake like Trout Bog and quadrillions of cells in a one-meter-depth layer of Lake Michigan. Assuming that the probability of sequencing is proportional to cell abundance and there are 500,000 cells per ml, a sequence from that population will not be detected 74% of the time and a single sequence will be detected 22% of the time from a sample with 150,000 sequences (slightly higher than any samples included in our meta-analysis), making the population likely to go unreported. Aquatic systems contain a systematically overlooked pool of diversity that may harbor organisms that immigrated from other habitats but have not become dominant in the system. These frequently overlooked low-abundance taxa may make disproportionately large contributions to ecosystem function (50) and could serve as a source of taxa available to take advantage of changing environmental conditions, akin to what Shade and colleagues (51) describe as “conditionally rare taxa.”

### Using 16S rRNA data sets to detect transitions.

There are several important caveats to consider when comparing microbial diversity between habitat types. First, we can only survey abundant, extant diversity. Analyzing 16S rRNA amplicon data sets gave us the benefit of deep sequencing relative to other approaches, but the 16S rRNA gene is not a good marker for differentiating closely related organisms (52). Marine and freshwater microorganisms classified as “shared” in our analyses may in fact exhibit substantial habitat-specific genome differentiation, and may be distinguishable as fine-scale sequence clusters based on the full-length 16S rRNA sequence or another housekeeping gene. Microbial community composition can also be affected by biases, including those resulting from DNA extraction method (53) and 16S rRNA gene primer set (28, 54). Shared taxa could potentially arise due to reagent contamination (55) or sample cross-contamination (56), but these issues are unlikely to explain our meta-analysis results, given that samples were processed and sequenced independently for each study.

### Summary.

Marine and freshwater systems are phylogenetically distinct, while at the same time harboring taxa that appear in both environments. Some taxonomic groups appear to be exclusive to marine or freshwater environments. At the same time, some taxonomic units appear in both habitat types; we identified 171 shared MED nodes across marine and freshwater habitats. It remains to be seen whether individual cells with marine-like or freshwater-like 16S rRNA resemble populations found in the other habitat genome-wide, or whether there is genomic mosaicism. Families at the extremes—lineages with a high degree of habitat-specific diversification or a large number of taxa found in both habitats—may serve as targets for future work investigating ecological plasticity and/or adaptations and the evolution of microbial lineages. There is clearly precedent and potential for marine and freshwater organisms to transition between habitats with different salinities and adapt to new environmental conditions, available resources, and interactions with neighboring organisms. A bank of near- or below-detection diversity, including cross-system immigrant populations, may contribute to community genomic diversity via horizontal gene transfer and exploit opportunities for niche expansion as environmental conditions change.

## MATERIALS AND METHODS

### 16S rRNA sequence processing.

We carried out a meta-analysis of marine and freshwater 16S rRNA gene sequencing data sets spanning the V4 region (Table 2; see also Table S2 in the supplemental material). For a data set to be included in our analysis, sequence reads needed to encompass bases 515 through 805 of the 16S rRNA gene. We augmented publicly available data sets with samples from the Laurentian Great Lakes sequenced by the Joint Genome Institute. Sequence processing was carried out using mothur v 1.38.1 unless otherwise noted (57). We merged paired sequence reads using make.contigs and quality filtered single reads using trim.seqs (window size = 50, minimum average quality score = 35). All sequences were then combined and processed following a modified version of the mothur MiSeq standard operating protocol accessed 27 September 2016 (58). Screening retained 200- to 300-bp sequences with no ambiguities and maximum homopolymer stretches fewer than 24 bases. Screened sequences were aligned to the Silva v128 reference alignment (59, 60), and chimeras were identified using UCHIME (61) and removed. Sequences were classified in mothur using Silva v128, and those identified as “Chloroplast,” “Mitochondria,” “unknown,” or “Eukaryota” were removed from the data set.

**TABLE 2:** 16S rRNA v4 region sequencing data sets included in the meta-analysis

No. of samples	Study system	Depth(s) sampled^*a*^	BioProject accession no. (reference)
Freshwater samples (45 total)			
11	Four Laurentian Great Lakes	Surface, DCL, deep	This study
2	Glacier Lake, NY	6 m, 14 m	PRJEB12903
1	JBL_J07_HES, Sweden	Integrated	PRJNA244610 (79)
1	Lake Keluke, China	Surface	PRJNA294836 (80)
1	Faselfad lakes, Austria	Integrated	PRJNA297573 (81)
14	Seven high-nutrient lakes, MI	Surface, deep	PRJNA304344 (82)
9	Five low-nutrient lakes, MI	Surface, deep	PRJNA304344 (82)
6	Three humic lakes, WI	Integrated epi, integrated hypo	PRJEB15148 (83)
Marine samples (32 total)			
1	Caribbean Sea	Surface	PRJEB10633 (28)
2	Coastal Red Sea (2 sites)	Surface	PRJNA279146 (54)
1	Drake Passage	Surface	PRJEB10633 (28)
12	Gulf of Mexico (3 sites)	Surface, multiple depths	PRJNA327040 (84)
1	Helgoland North Sea	Surface	PRJNA266669 (85)
1	Long Island Sound	Surface	PRJEB10633 (28)
2	North Pacific	Surface, 100 m	PRJEB10633 (28)
8	San Pedro Ocean Time Series (2 dates: April, July 2013)	Surface, multiple depths	PRJEB10633 (28)
2	Sargasso Sea (2 sites)	Surface, 200 m	PRJEB10633 (28)
1	Tropical Western Atlantic Ocean	40 m	PRJEB10633 (28)
1	Weddell Sea	Surface	PRJEB10633 (28)

a Abbreviations: DCL, deep chlorophyll layer; epi, epilimnion; hypo, hypolimnion.

10.1128/mSystems.00232-18.10 TABLE S2 Additional information on 16S rRNA tag sequencing data sets compiled for the meta-analysis. Download Table S2, PDF file, 0.1 MB. Copyright © 2018 Paver et al. 2018 Paver et al. This content is distributed under the terms of the Creative Commons Attribution 4.0 International license.

We used two approaches to cluster similar sequences. First, we implemented minimum entropy decomposition (MED), a method that employs Shannon entropy to partition sequences into taxonomic units referred to as “nodes” using information-rich nucleotide positions and ignoring stochastic variation (27). We ran MED with a minimum substantive abundance of 10 sequences and 4 discriminant locations. Second, to quantify taxon relatedness based on absolute sequence identity, we implemented direct comparisons for all sequences within each phylum (proteobacterial class). We calculated pairwise sequence distances and used farthest-neighbor clustering to cluster sequences at all possible sequence identity cutoff values, from 100% identity down to the level where all sequences collapse into a single cluster, at a precision of 1,000. For groups with distance matrices too large to process all sequences together (*Alphaproteobacteria*, *Bacteroidetes*, *Betaproteobacteria*, *Gammaproteobacteria*), cluster.split was implemented at the order level (taxonomic level 4) to group sequences into taxonomic units at cutoff values from unique down to 0.30. Classification-based cluster splitting was not a feasible approach for *Actinobacteria*, so pre.cluster was run on sequences prior to calculating furthest-neighbor clusters.

### Statistical and phylogenetic comparisons of marine and freshwater data sets.

We compared marine and freshwater samples and sequences at the levels of taxonomic classification, MED nodes, and sequence identity using R version 3.3.2 (R Core Team, 2016). Phyla (proteobacterial classes) that were differentially abundant in marine versus freshwater samples were identified by testing for differences in the log_2_ fold change using a parametric Wald test implemented by DESeq2 (62).

To quantify phylogenetic distance between marine and freshwater taxa, we first generated a maximum-likelihood tree from mothur-aligned sequences using the GTRGAMMA model in RaxML v7.7.9 (63). To visualize subtrees and calculate UniFrac distances for specific groups, bacterial trees were rooted with a Marine Group I archaeal sequence (A000001667) using the APE R package (64). Trees were visualized using the interactive Tree Of Life (65). Sequences were rarefied, and data were subset for subsequent analyses using the phyloseq package (66). We rarefied samples to even depth (9,827 sequences/sample) and calculated unweighted UniFrac distances for each pair of samples using GUniFrac (67). We tested the significance of UniFrac distances between marine and freshwater samples using permutational multivariate analysis of variance (PerMANOVA) as implemented by the Adonis function in the Vegan package (68). The same approach was used to calculate UniFrac values for each phylum and proteobacterial class that was observed in at least three marine and three freshwater samples and contained at least five MED nodes. Samples containing fewer than 500 sequences for a given phylum or class were removed from the analysis. We combined all sequences from each habitat and calculated unweighted UniFrac distances for phyla, proteobacterial classes, orders and families using GUniFrac following sequence rarefaction to even depth. To test the significance of calculated UniFrac values for each phylum and proteobacterial class, unweighted UniFrac values were calculated for 1,000 independent swap randomizations of the presence-absence sample matrix generated by the randomizeMatrix function in the Picante package (69). Using these distances as a null distribution, one-sample z tests were conducted to test the null hypothesis that the phylogenetic tree is not grouped by habitat. A Bonferroni correction was implemented as a conservative measure to account for multiple testing in determining significance.

We identified “shared nodes” as MED nodes observed in the unrarefied sequence set for at least one marine and at least one freshwater sample. For each phylum (proteobacterial class) containing more than five nodes observed in both marine and freshwater samples (i.e., shared nodes), we generated accumulation curves to visualize the number of shared MED nodes as a function of the number of freshwater or marine sampling sites using the specaccum function in the vegan package (68).

To compare sequence clusters generated at each sequence identity cutoff, we calculated Jaccard distances for pairs of unrarefied samples (one marine and one freshwater) for each phylum (proteobacterial class). The Jaccard distance is calculated as 1 minus the intersection of two samples (i.e., the number of shared units) divided by the union of two samples (i.e., the total number of units); a Jaccard distance of 1 means that no taxa are shared between samples. The sequence identity cutoff value at which marine-freshwater sample pairs first contain shared taxa (Jaccard distance < 1) was summarized for each phylum/class using boxplots.

### Metagenomic evidence of non-LD12 SAR11 in the Great Lakes.

To test whether there is genome-wide evidence beyond the 16S rRNA locus for non-LD12 SAR11 cells in the Great Lakes, we identified reads that mapped with high confidence to non-LD12 SAR11 in a SAR11/LD12 reference phylogeny. Briefly, we analyzed merged pairs of metagenome sequencing reads from samples collected from the surface of each of the Great Lakes in spring 2012 (IMG taxon object IDs: 3300005580, 3300005582, 3300005583, 3300005584, 3300005585) as well as a marine sample from the Tara Oceans project collected from the North Atlantic Ocean Westerlies Biome near Bermuda for comparison (accession number ERR599123) (70). Sequence reads from each metagenome were searched against the nr protein database (71; downloaded 13 April 2017) using Diamond v. 0.8.18.80 (72), and sequences whose best hit matched the *Pelagibacterales* family were identified using Krona Tools v. 2.7 (73) and extracted. These putative *Pelagibacterales* reads were then mapped to a database of SAR11/LD12 protein clusters using a translated query-protein subject (blastx) BLAST v. 2.2.28 (74) with E value <0.001 and alignment length >60 amino acids (>50 amino acids for the Tara Oceans sample since sequence reads were shorter). We set a liberal E value threshold for this reciprocal BLAST step in order to sensitively map short metagenomic fragments to protein clusters; in practice, the median E value for mapping reads in this step was 2 × 10^−47^, with 99% of query reads yielding E values less than 2 × 10^−15^ and 99.7% of reads yielding E values less than 1 × 10^−6^. Our protein cluster database was constructed from publicly available SAR11 and LD12 genomes (Table 3) using all-vs-all blastp and MCL clustering (75) as implemented by Anvi’o v. 2.4.0 (76). We focused our analysis on putative core protein clusters found in single copy in at least 6 SAR11 genomes and 3 LD12 genomes; notably, all but one LD12 genome derive from single-cell genome amplification and sequencing and are therefore incomplete. For each protein cluster, we backtranslated amino acid alignments, generated by MUSCLE v. 3.8 (77) within Anvi’o, to nucleotide alignments. We then used RAxML v. 7.2.6 with the GTRGAMMA model to generate a maximum likelihood tree for each protein cluster based on its corresponding nucleotide alignment (63). Metagenomic reads that mapped to each protein cluster by blastx were aligned to the cluster’s reference nucleotide alignment using HMMER v 3.1b2 (hmmer.org). Reads were then classified taxonomically using pplacer with the -p flag to calculate prior probabilities and guppy classify using the -pp flag to use posterior probability for the pplacer classifier criteria v1.1.alpha19-0-g807f6f3 (78). The reference package for taxonomy classification was generated using taxtastic v 0.5.4 (http://fhcrc.github.io/taxtastic/index.html). We analyzed the resulting databases using the R package BoSSA v2.1 (https://cran.r-project.org/web/packages/BoSSA/index.html).

**TABLE 3:** SAR11 and LD12 genomes included in pangenomic analysis

Genome name	SAR11 clade	Classification	GenBank accession no.
Alphaproteobacterium HIMB114	IIIa	SAR11	NZ_ADAC02000001
Alphaproteobacterium HIMB59	V	SAR11	NC_018644
“*Candidatus* Pelagibacter” sp. HTCC7211	Ia.2	SAR11	ABVS00000000
“*Candidatus* Pelagibacter” sp. IMCC9063	IIIa	SAR11	NC_015380
“*Candidatus* Pelagibacter ubique” HIMB058	II	SAR11	ATTF01000000
“*Candidatus* Pelagibacter ubique” HIMB083	Ia.2	SAR11	AZAL00000000
“*Candidatus* Pelagibacter ubique” HTCC1062	Ia.1	SAR11	NC_007205
“*Candidatus* Pelagibacter ubique” HTCC8051	Ia.2	SAR11	AWZY00000000
SCGC AAA280-B11	IIIb	LD12	AQUH00000000
SCGC AAA027-C06	IIIb	LD12	AQPD00000000
SCGC AAA028-C07	IIIb	LD12	ATTB01000000
SCGC AAA028-D10	IIIb	LD12	AZOF00000000
SCGC AAA280-P20	IIIb	LD12	AQUE00000000
SCGC AAA023-L09	IIIb	LD12	ATTD01000000
SCGC AAA027-L15	IIIb	LD12	AQUG00000000
SCGC AAA487-M09	IIIb	LD12	ATTC00000000
SCGC AAA024-N17	IIIb	LD12	AQZA00000000
SCGC AAA280-P20	IIIb	LD12	AQUE00000000
“*Candidatus* Fonsibacter ubiquis” LSUCC0530	IIIb	LD12	NZ_CP024034
Lake_Baikal_MAG	Unknown	SAR11	NSIJ01000001

### Data availability.

Laurentian Great Lakes sequences are available on the Joint Genome Institute’s genome data portal (http://genome.jgi.doe.gov/; project identifiers, 1045074 and 1045077). R code and associated data files are available at https://bitbucket.org/greatlakes/marine_fw_meta.git. Intermediate data files referenced in code but too large to store on bitbucket are available on figshare at https://doi.org/10.6084/m9.figshare.7180649.v1.
