# Supplementary material for: Reevaluating the Salty Divide: Phylogenetic Specificity of Transitions between Marine and Freshwater Systems
Source: mSystems. 2018 Nov 13;3(6):e00232-18. doi: 10.1128/mSystems.00232-18 (PMC6234284; doi:10.1128/mSystems.00232-18)
Supplement: TABLE S1 [file sys006182289st1.pdf]

Table S1. UniFrac distance calculated between marine and freshwater sequences for each family.

phylum	family	unifrac distance
Actinobacteria	PeM15_fa	0.51
Gammaproteobacteria	Vibrionaceae	0.52
Gammaproteobacteria	Chromatiaceae	0.52
Bacteroidetes	Unknown_Family	0.53
Gammaproteobacteria	Unknown_Family	0.53
Verrucomicrobia	Unknown_Family	0.53
Actinobacteria	Mycobacteriaceae	0.54
Gammaproteobacteria	Moraxellaceae	0.59
Alphaproteobacteria	Caulobacteraceae	0.61
Gammaproteobacteria	Spongiibacteraceae	0.64
Actinobacteria	uncultured	0.64
Alphaproteobacteria	uncultured	0.64
Bacteroidetes	uncultured	0.64
Gammaproteobacteria	uncultured	0.64
Actinobacteria	Sporichthyaceae	0.65
Bacteroidetes	Cryomorphaceae	0.66
Bacteroidetes	Chitinophagaceae	0.67
Gammaproteobacteria	OM182_clade	0.67
Alphaproteobacteria	Rhodobacteraceae	0.68
Gammaproteobacteria	Oceanospirillaceae	0.70
Alphaproteobacteria	Hyphomonadaceae	0.71
Alphaproteobacteria	Rickettsiaceae	0.71
Verrucomicrobia	DEV007	0.73
Deltaproteobacteria	SAR324_clade(Marine_group_B)_fa	0.73
Alphaproteobacteria	Sphingomonadaceae	0.73
Deltaproteobacteria	Bacteriovoracaceae	0.75
Gammaproteobacteria	Halieaceae	0.75
Bacteroidetes	Flavobacteriaceae	0.77
Bacteroidetes	NS11-12_marine_group	0.77
Betaproteobacteria	Comamonadaceae	0.79
Chloroflexi	JG30-KF-CM66_fa	0.79
Verrucomicrobia	Verrucomicrobiaceae	0.80
Actinobacteria	Microbacteriaceae	0.81
Gammaproteobacteria	Oceanospirillales_unclassified	0.81
Betaproteobacteria	Burkholderiaceae	0.82
Bacteroidetes	Saprospiraceae	0.82

Table S1 contd. UniFrac distance calculated between marine and freshwater sequences for each family.

phylum	family	unifrac distance
Cyanobacteria	FamilyI	0.84
Gammaproteobacteria	Gammaproteobacteria_unclassified	0.86
Planctomycetes	Planctomycetaceae	0.86
Chloroflexi	Anaerolineaceae	0.86
Alphaproteobacteria	AT-s3-44	0.86
Planctomycetes	Phycisphaeraceae	0.87
Betaproteobacteria	Methylophilaceae	0.89
Betaproteobacteria	Betaproteobacteria_unclassified	0.91
Verrucomicrobia	OPB35_soil_group_fa	0.91
Bacteroidetes	Sphingobacteriales_unclassified	0.92
Bacteroidetes	Bacteroidetes_unclassified	0.92
Betaproteobacteria	Alcaligenaceae	0.93
Bacteroidetes	NS9_marine_group	0.93
Alphaproteobacteria	Rhodospirillales_unclassified	0.93
Alphaproteobacteria	Rhodospirillaceae	0.93
Alphaproteobacteria	Alphaproteobacteria_unclassified	0.93
Deltaproteobacteria	Deltaproteobacteria_unclassified	0.95
Deltaproteobacteria	Bdellovibrionaceae	0.95
Deltaproteobacteria	Blfdi19	0.95
Cyanobacteria	ML635J-21_fa	0.96
Gammaproteobacteria	Coxiellaceae	0.96
Actinobacteria	Acidimicrobiaceae	0.97
Deltaproteobacteria	Oligoflexaceae	0.98
Gammaproteobacteria	Pseudoalteromonadaceae	1.00
Betaproteobacteria	Hydrogenophilaceae	1.00
Gammaproteobacteria	KI89A_clade_fa	1.00
