# Supplementary figures and images for: Reevaluating the Salty Divide: Phylogenetic Specificity of Transitions between Marine and Freshwater Systems
Source: mSystems. 2018 Nov 13;3(6):e00232-18. doi: 10.1128/mSystems.00232-18 (PMC6234284; doi:10.1128/mSystems.00232-18)

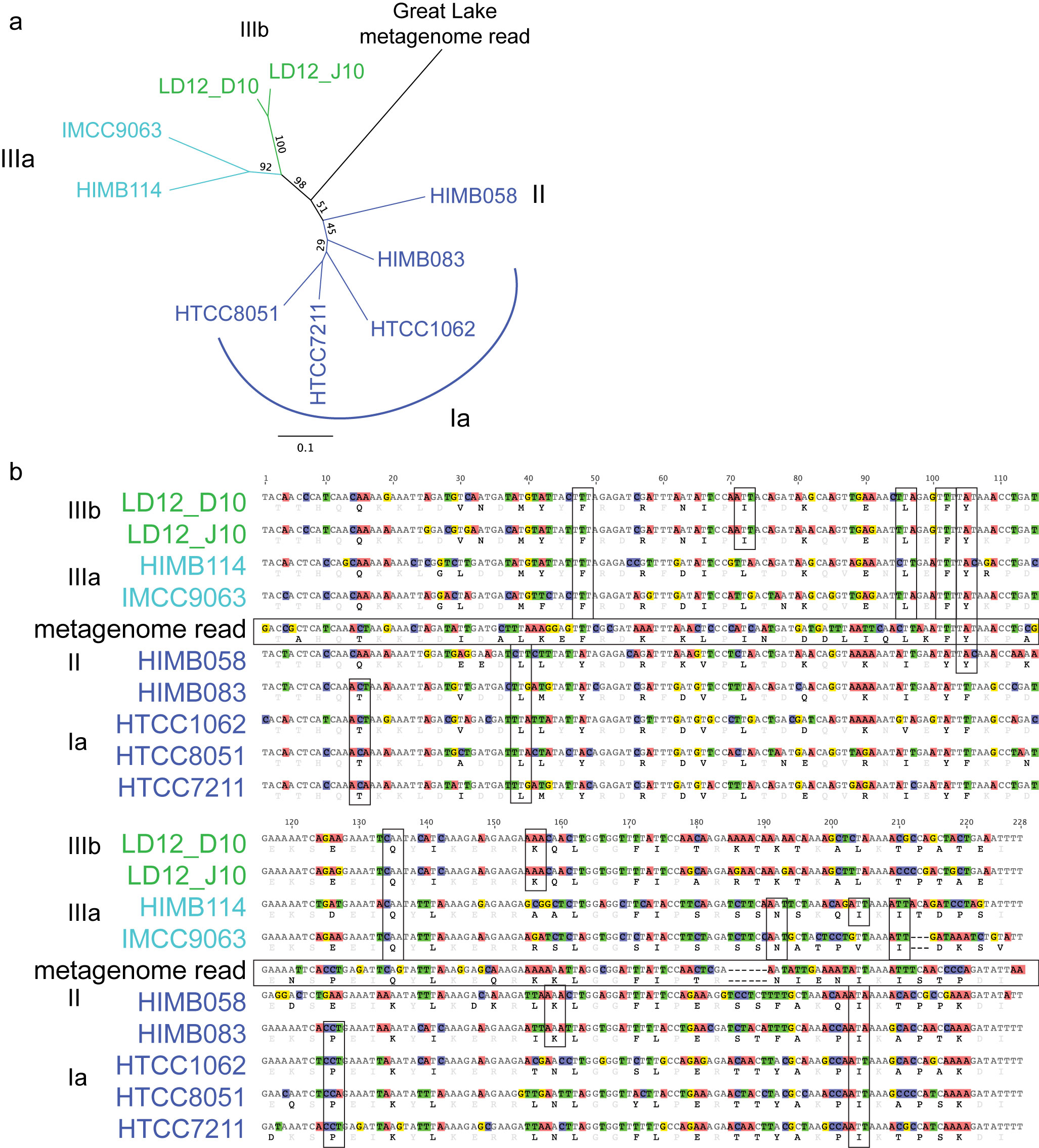

Supplement: FIG S8 [file sys006182289sf8.jpg]
