# Supplementary material for: Reevaluating the Salty Divide: Phylogenetic Specificity of Transitions between Marine and Freshwater Systems
Source: mSystems. 2018 Nov 13;3(6):e00232-18. doi: 10.1128/mSystems.00232-18 (PMC6234284; doi:10.1128/mSystems.00232-18)
Supplement: TABLE S2 [file sys006182289st2.pdf]

Table S2. Additional information on 16S rRNA tag sequencing datasets compiled for the meta-analysis.

| Study system | Sample name | Depth | Habitat type | Platform | F_primer | R_primer | Data base | Sample accession/ JGI project | citation |
|---|---|---|---|---|---|---|---|---|---|
| Freshwater samples (45 total) | | | | | | | | | |
| Great Lakes | ON55M_DCL_Aug2013 | DCL | oligomesotrophic | MiSeq | 515F-C | 806R-H | JGI | GreLakitagplate2 | Our Study |
| Great Lakes | MI41M_DCL_Aug2013 | DCL | oligotrophic | MiSeq | 515F-C | 806R-H | JGI | GreLakitagplate2 | Our Study |
| Great Lakes | SU08M_DCL_Aug2013 | DCL | oligotrophic | MiSeq | 515F-C | 806R-H | JGI | GreLakitagplate2 | Our Study |
| Great Lakes | ER91M_Bminus_Aug2013 | Bminus[D] | mesotrophic | MiSeq | 515F-C | 806R-H | JGI | GreLakitagplate2 | Our Study |
| Great Lakes | ON55M_B10_Aug2013 | B10[D] | oligomesotrophic | MiSeq | 515F-C | 806R-H | JGI | GreLakitagplate2 | Our Study |
| Great Lakes | MI41M_B10_Aug2013 | B10[D] | oligotrophic | MiSeq | 515F-C | 806R-H | JGI | GreLakitagplate2 | Our Study |
| Great Lakes | SU08M_B10_Aug2013 | B10[D] | oligotrophic | MiSeq | 515F-C | 806R-H | JGI | GreLakitagplate2 | Our Study |
| Great Lakes | ER91M_SRF_Aug2013 | Surface[S] | mesotrophic | MiSeq | 515F-C | 806R-H | JGI | GreLakitagplate2 | Our Study |
| Great Lakes | ON55M_SRF_Aug2013 | Surface[S] | oligomesotrophic | MiSeq | 515F-C | 806R-H | JGI | GreLakitagplate2 | Our Study |
| Great Lakes | MI41M_SRF_Aug2013 | Surface[S] | oligotrophic | MiSeq | 515F-C | 806R-H | JGI | GreLakitagplate2 | Our Study |
| Great Lakes | SU08M_SRF_Aug2013 | Surface[S] | oligotrophic | MiSeq | 515F-C | 806R-H | JGI | GreLakitagplate2 | Our Study |
| Glacier Lake, NY | glacier_lake_6m | 6m[S] | meromictic | unk | unk | unk | ENA | ERR1299038 | (PRJEBI2903) |
| Glacier Lake, NY | glacier_lake_14m | 14m[D] | meromictic | unk | unk | unk | ENA | ERR1299042 | (PRJEBI2903) |
| Swedish Lake | JBL_J07_HES | integrated | oligotrophic | 454 | 341F | 805R | ENA | SRR444593 | Logue et al. 2012 |
| Lake Keluke, China | Lake_Keluke_1gl_salinity | Surface[S] | freshwater | MiSeq | 515F-C | 806R-H | ENA | SRR2310185 | Zhong et al. 2016 |
| Faselfad lakes | Faselfad BACK-ALP | unknown | | 454 | unknown | unknown | ENA | SRR2545928 | Peter et al. 2016 |
| MI Lakes | BAKE1 | Surface[S] | high | MiSeq | 515F-C | 806R-H | ENA | SRR2962903 | Schmidt et al. 2016 |
| MI Lakes | BASH1 | Hypolimnion[D] | high | MiSeq | 515F-C | 806R-H | ENA | SRR2962906 | Schmidt et al. 2016 |
| MI Lakes | BRIE1 | Surface[S] | high | MiSeq | 515F-C | 806R-H | ENA | SRR2962910 | Schmidt et al. 2016 |
| MI Lakes | BRIH1 | Hypolimnion[D] | high | MiSeq | 515F-C | 806R-H | ENA | SRR2962914 | Schmidt et al. 2016 |
| MI Lakes | BSTE1 | Surface[S] | high | MiSeq | 515F-C | 806R-H | ENA | SRR2962919 | Schmidt et al. 2016 |
| MI Lakes | BSTH1 | Hypolimnion[D] | high | MiSeq | 515F-C | 806R-H | ENA | SRR2962922 | Schmidt et al. 2016 |
| MI Lakes | GULE1 | Surface[S] | low | MiSeq | 515F-C | 806R-H | ENA | SRR2962927 | Schmidt et al. 2016 |
| MI Lakes | GULE2 | Surface[S] | low | MiSeq | 515F-C | 806R-H | ENA | SRR2962930 | Schmidt et al. 2016 |
| MI Lakes | GULH2 | Hypolimnion[D] | low | MiSeq | 515F-C | 806R-H | ENA | SRR2962934 | Schmidt et al. 2016 |
| MI Lakes | BAKH1 | Hypolimnion[D] | high | MiSeq | 515F-C | 806R-H | ENA | SRR2962939 | Schmidt et al. 2016 |
| MI Lakes | LEEH1 | Surface[S] | low | MiSeq | 515F-C | 806R-H | ENA | SRR2962940 | Schmidt et al. 2016 |
| MI Lakes | LEEH2 | Hypolimnion[D] | low | MiSeq | 515F-C | 806R-H | ENA | SRR2962942 | Schmidt et al. 2016 |
| MI Lakes | LONE1 | Surface[S] | low | MiSeq | 515F-C | 806R-H | ENA | SRR2962945 | Schmidt et al. 2016 |
| MI Lakes | LONH1 | 7m[D] | low | MiSeq | 515F-C | 806R-H | ENA | SRR2962947 | Schmidt et al. 2016 |
| MI Lakes | PAYE1 | Surface[S] | high | MiSeq | 515F-C | 806R-H | ENA | SRR2962950 | Schmidt et al. 2016 |
| MI Lakes | PAYH1 | Hypolimnion[D] | high | MiSeq | 515F-C | 806R-H | ENA | SRR2962955 | Schmidt et al. 2016 |
| MI Lakes | SHEE1 | Surface[S] | high | MiSeq | 515F-C | 806R-H | ENA | SRR2962959 | Schmidt et al. 2016 |
| MI Lakes | SHEH1 | bottom | high | MiSeq | 515F-C | 806R-H | ENA | SRR2962965 | Schmidt et al. 2016 |
| MI Lakes | SIXE2 | Surface[S] | low | MiSeq | 515F-C | 806R-H | ENA | SRR2962970 | Schmidt et al. 2016 |
| MI Lakes | SIXH1 | Hypolimnion[D] | low | MiSeq | 515F-C | 806R-H | ENA | SRR2962972 | Schmidt et al. 2016 |
| MI Lakes | WINE1 | Surface[S] | high | MiSeq | 515F-C | 806R-H | ENA | SRR2962976 | Schmidt et al. 2016 |
| MI Lakes | WINH1 | Hypolimnion[D] | high | MiSeq | 515F-C | 806R-H | ENA | SRR2962980 | Schmidt et al. 2016 |
| MI Lakes | BASE1 | Surface[S] | high | MiSeq | 515F-C | 806R-H | ENA | SRR2962984 | Schmidt et al. 2016 |
| Humic Lakes | CBH18Jul08_3 | integrated_ hypolimnion[D] | dystrophic | MiSeq | 515F-C | 806R-H | JGI | CrystalBogitags | Linz et al. 2017 |
| Humic Lakes | NSBH16JUL13 | integrated_ hypolimnion[D] | dystrophic | MiSeq | 515F-C | 806R-H | JGI | CrystalBogitags | Linz et al. 2017 |

| Study system | Sample name | Depth | Habitat type | Platform | F_primer | R_primer | Data base | Sample accession/ JGI project | citation |
|---|---|---|---|---|---|---|---|---|---|
| | | | | Freshwater continued | | | | | |
| Humic Lakes | TBH08AUG13 | integrated_ hypolimnion[D] | dystrophic | MiSeq | 515F-C | 806R-H | JGI | CrystalBogitags | Linz et al. 2017 |
| Humic Lakes | CBE18Jul08_3 | integrated_ epilimnion[S] | dystrophic | MiSeq | 515F-C | 806R-H | JGI | CrystalBogitags | Linz et al. 2017 |
| Humic Lakes | NSBE16JUL13 | integrated_ epilimnion[S] | dystrophic | MiSeq | 515F-C | 806R-H | JGI | CrystalBogitags | Linz et al. 2017 |
| Humic Lakes | TBE08AUG13 | integrated_ epilimnion[S] | dystrophic | MiSeq | 515F-C | 806R-H | JGI | CrystalBogitags | Linz et al. 2017 |
| | | | | Marine samples (32 total) | | | | | |
| Caribbean Sea | Carribean Sea | Surface[S] | pelagic | MiSeq | 515F-Y | 806R-H | ENA | ERR995952 | Parada et al. 2016 |
| Drake Passage | Drake Passage | Surface[S] | pelagic | MiSeq | 515F-Y | 806R-H | ENA | ERR995950 | Parada et al. 2016 |
| Long Island Sound | long_island_sound_SRF | Surface[S] | coastal | MiSeq | 515F-C | 806R-H | ENA | ERR995890 | Parada et al. 2016 |
| North Pacific | north_pacific_100m | 100m[D] | pelagic | MiSeq | 515F-Y | 806R-H | ENA | ERR995978 | Parada et al. 2016 |
| North Pacific | north_pacific_SRF | Surface[S] | pelagic | MiSeq | 515F-Y | 806R-H | ENA | ERR995976 | Parada et al. 2016 |
| SPOTS | SPOT_4_2013_DCM | DCL | pelagic | MiSeq | 515F-C | 806R-H | ENA | ERR995866 | Parada et al. 2016 |
| SPOTS | SPOT_07_18_2013_150m | 150m[D] | pelagic | MiSeq | 515F-Y | 806R-H | ENA | ERR995880 | Parada et al. 2016 |
| SPOTS | SPOT_07_18_2013_890m | 890m[D] | pelagic | MiSeq | 515F-Y | 806R-H | ENA | ERR995888 | Parada et al. 2016 |
| SPOTS | SPOT_4_2013_150m | 150m[D] | pelagic | MiSeq | 515F-C | 806R-H | ENA | ERR995846 | Parada et al. 2016 |
| SPOTS | SPOT_4_2013_500m | 500m[D] | pelagic | MiSeq | 515F-C | 806R-H | ENA | ERR995850 | Parada et al. 2016 |
| SPOTS | SPOT_4_2013_890m | 890m[D] | pelagic | MiSeq | 515F-C | 806R-H | ENA | ERR995862 | Parada et al. 2016 |
| SPOTS | SPOT_07_18_2013_5m | 5m[S] | pelagic | MiSeq | 515F-Y | 806R-H | ENA | ERR995886 | Parada et al. 2016 |
| SPOTS | SPOT_4_2013_5m | 5m[S] | pelagic | MiSeq | 515F-C | 806R-H | ENA | ERR995854 | Parada et al. 2016 |
| Sargasso Sea | sargasso_sea_st2 | 200m[D] | pelagic | MiSeq | 515F-C | 806R-H | ENA | ERR995931 | Parada et al. 2016 |
| Sargasso Sea | sargasso_sea_st1 | Surface[S] | pelagic | MiSeq | 515F-C | 806R-H | ENA | ERR995960 | Parada et al. 2016 |
| Tropical Western Atlantic Ocean | Tropical Western Atlantic Ocean | 40m | pelagic | MiSeq | 515F-Y | 806R-H | ENA | ERR995974 | Parada et al. 2016 |
| Weddell Sea | weddell_sea | unknown | pelagic | MiSeq | 515F-Y | 806R-H | ENA | ERR995993 | Parada et al. 2016 |
| Coastal Red Sea | Coastal Red Sea (w1) | Surface[S] | coastal | MiSeq | 515F-C | 806R-H | ENA | SRR1927833 | Apprill et al. 2015 |
| Coastal Red Sea | Coastal Red Sea (w23) | Surface[S] | coastal | MiSeq | 515F-C | 806R-H | ENA | SRR1927842 | Apprill et al. 2015 |
| Hegoland North Sea | Hegoland_north_sea_LTE R | Surface[S] | coastal | MiSeq | 515F-C | 806R-H | ENA | SRR2104389 | Teeling et al. 2016 |
| Gulf of Mexico | DWH.P3.1 | Surface[S] | coastal | MiSeq | 515F-C | 806R-H | ENA | SRR3731313 | Mason et al. 2016 |
| Gulf of Mexico | DWH.P3.25 | 25m | coastal | MiSeq | 515F-C | 806R-H | ENA | SRR3731314 | Mason et al. 2016 |
| Gulf of Mexico | DWH.P3.50 | 50m | coastal | MiSeq | 515F-C | 806R-H | ENA | SRR3731315 | Mason et al. 2016 |
| Gulf of Mexico | DWH.P3.75 | 75m[D] | coastal | MiSeq | 515F-C | 806R-H | ENA | SRR3731316 | Mason et al. 2016 |
| Gulf of Mexico | DWH.P3.200 | 200m[D] | coastal | MiSeq | 515F-C | 806R-H | ENA | SRR3731317 | Mason et al. 2016 |
| Gulf of Mexico | DWH.P3.300 | 300m[D] | coastal | MiSeq | 515F-C | 806R-H | ENA | SRR3731318 | Mason et al. 2016 |
| Gulf of Mexico | LC.P4.1 | Surface | coastal | MiSeq | 515F-C | 806R-H | ENA | SRR3731319 | Mason et al. 2016 |
| Gulf of Mexico | LC.P4.25 | 25m | coastal | MiSeq | 515F-C | 806R-H | ENA | SRR3731320 | Mason et al. 2016 |
| Gulf of Mexico | LC.P4.50 | 50m | coastal | MiSeq | 515F-C | 806R-H | ENA | SRR3731322 | Mason et al. 2016 |
| Gulf of Mexico | LC.P4.90 | 90m[D] | coastal | MiSeq | 515F-C | 806R-H | ENA | SRR3731323 | Mason et al. 2016 |
| Gulf of Mexico | LC.P4.150 | 150m[D] | coastal | MiSeq | 515F-C | 806R-H | ENA | SRR3731324 | Mason et al. 2016 |
| Gulf of Mexico | C.P5.1 | Surface[S] | coastal | MiSeq | 515F-C | 806R-H | ENA | SRR3731330 | Mason et al. 2016 |

[S] included in the “surface” group for depth-specific analyses

[D] included in the “deep” group for depth-specific analyses
